# An unusual cause of upper gastrointestinal bleeding

**DOI:** 10.1093/jcag/gwae045

**Published:** 2024-11-05

**Authors:** Tony He, Gary May, Christopher Teshima

**Affiliations:** Department of Gastroenterology, St. Michael’s Hospital, Toronto, Ontario; Department of Gastroenterology, St. Michael’s Hospital, Toronto, Ontario; Department of Gastroenterology, St. Michael’s Hospital, Toronto, Ontario

**Keywords:** gastrointestinal bleeding, oesophageal stricture, oesophageal stent

A 71-year-old gentleman presented with chronic dysphagia complicated by severe malnutrition secondary to a 1 cm length, 8 mm diameter recalcitrant distal oesophageal peptic stricture. This was on a background of spina bifida, alcohol dependence disorder, and neurogenic bladder requiring self-catheterization.

Between 2016 and 2023, the patient underwent serial dilatations with limited clinical benefit. He was then referred for placement of an oesophageal metal stent. A 14 mm wide × 30 mm long Hanaro Plumber metal stent was then placed (Figure 1).

**Figure 1.**
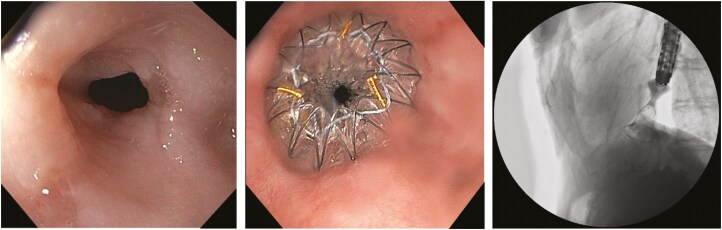
Endoscopic views of a recalcitrant benign distal oesophageal stricture before and after deployment of a 14 mm wide × 30 mm long Hanaro Plumber metal stent. Fluoroscopic confirmation of successful metal stent deployment.

Four days post-operatively, the patient returned with nausea and vomiting. Computed tomography (CT) did not identify a cause for his symptoms; however, the distance between the aorta and the oesophageal metal stent was 3.2 mm (Figure 2). Fourteen days post-operatively, the patient developed haematemesis complicated by severe hypotension requiring massive transfusion activation. An EGD revealed a large clot arising from within the oesophageal metal stent without a clear treatable target. A CT angiogram confirmed an aortic injury with pseudoaneurysm formation and active extravasation into the proximal stomach (Figure 3). Emergent thoracic endovascular repair was undertaken (Figure 4) and hemostasis was achieved. Despite successful hemostasis, the patient passed away 2 days later from multi-organ failure.

**Figure 2.**
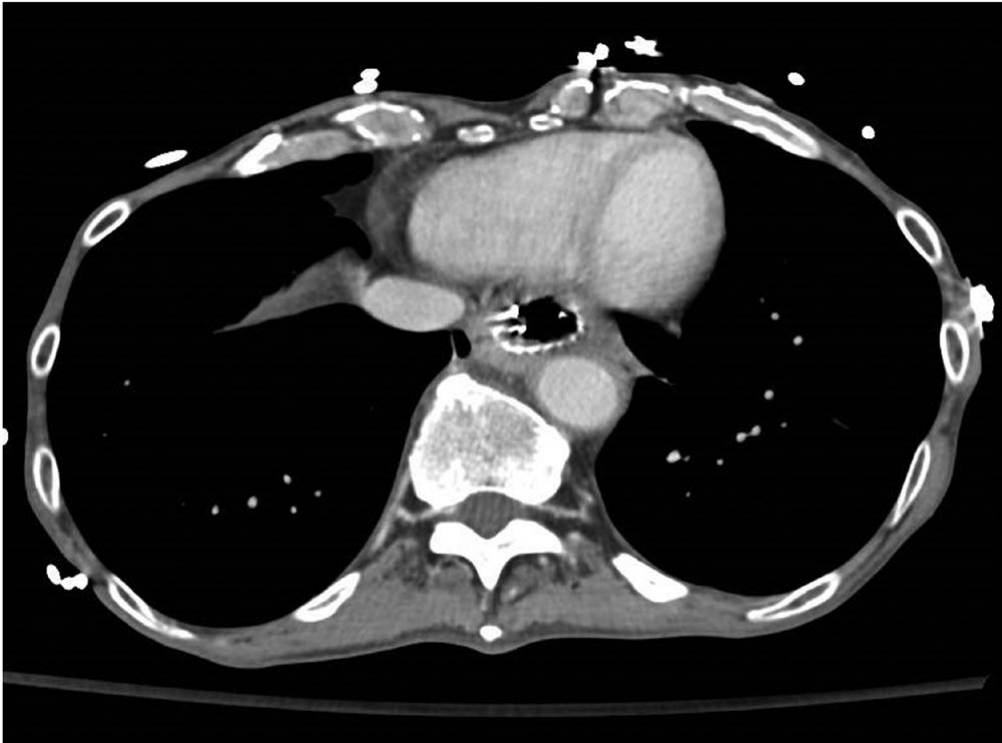
Thoracic and abdominal computed tomography confirming no oesophageal stent migration or cause for the patient’s nausea and vomiting.

**Figure 3.**
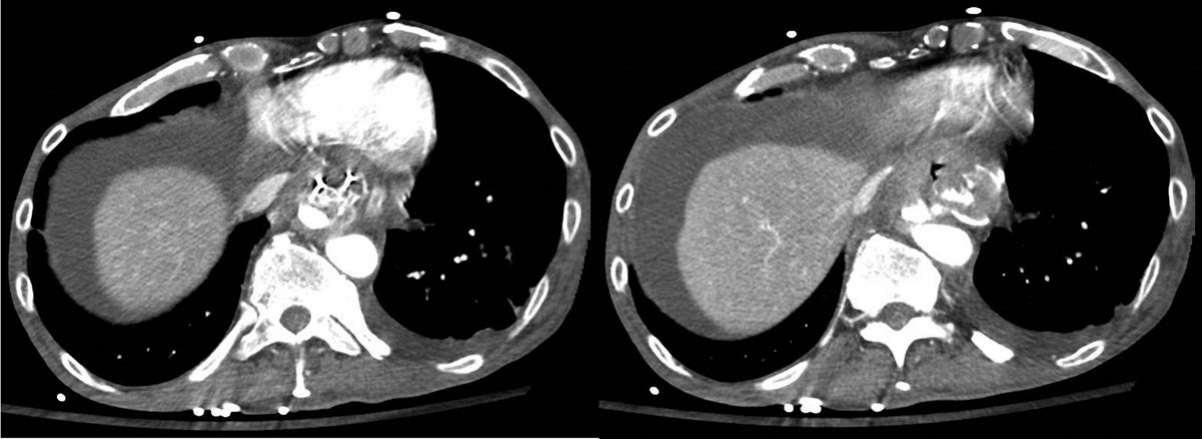
Peri-aortic haematoma and lobulated pseudoaneurysm; oesophageal stent and aorta. Nipple-like outpouching from aortic injury with active arterial extravasation.

**Figure 4.**
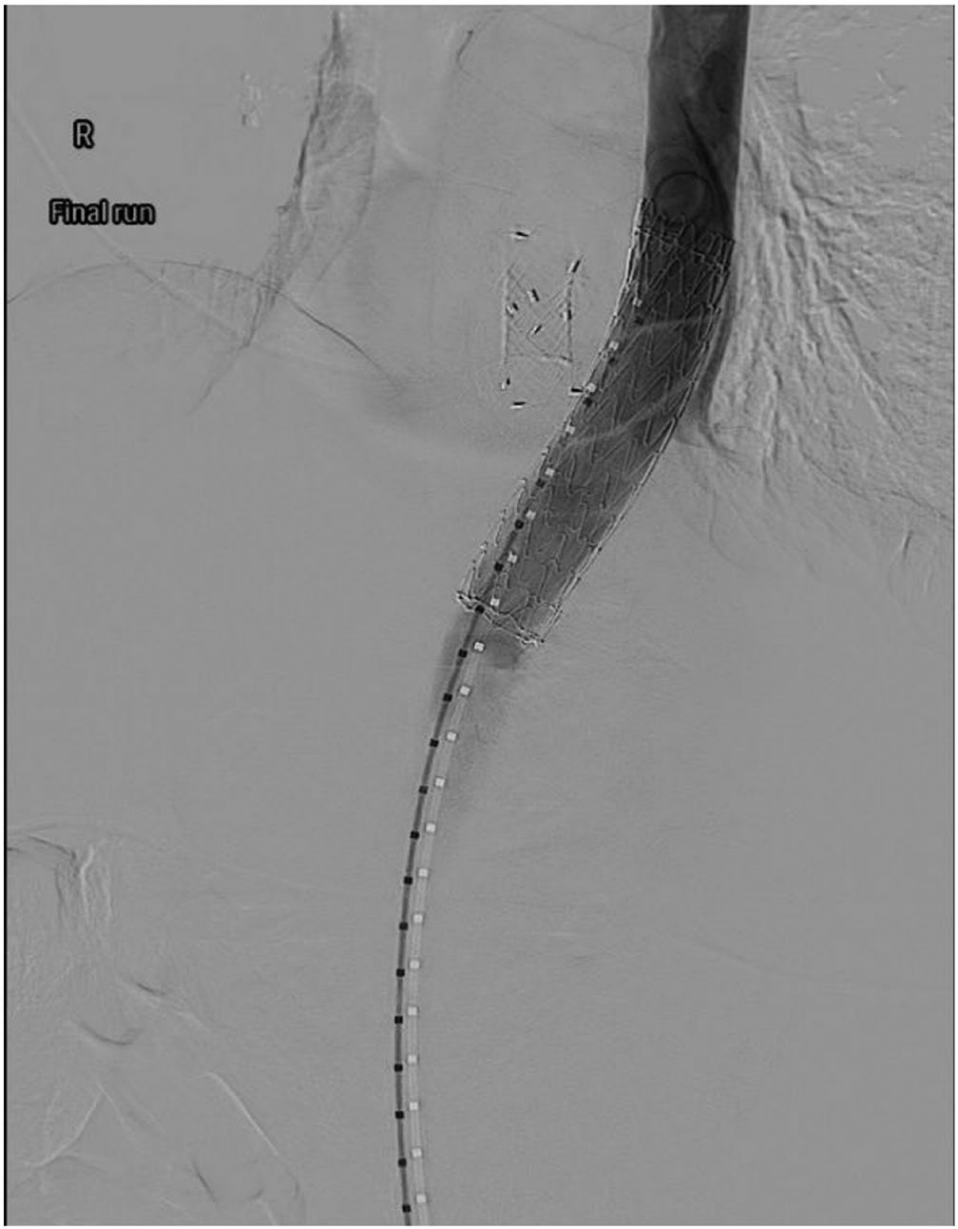
Thoracic endovascular aortic repair with successful placement of a 26 mm × 10 cm thoracic endograft with no contrast leak. The oesophageal stent is seen adjacent.

There are a small number of case reports that describe oesophageal stent-related aorto-oesophageal fistula (AEF) formation.^1–4^ Proposed risk factors include prior repeated dilatations, radiotherapy, or proximal stricture location.^3,4^ In our case, the bi-flanged metal stent may have also increased the risk of AEF formation secondary to local compression of the oesophageal mucosa. CT angiogram is the gold standard for diagnosis.^5^ AEF-associated mortality is approximately 75% with intervention and 100% without intervention.^5^ Prompt recognition of AEF to facilitate urgent intervention is therefore paramount.

## Supplementary Material

gwae045_Supplementary_Material

## Data Availability

The data are only of one patient. There are no other data associated with this manuscript.
